# Viszeralchirurgie im Massenanfall bei Katastrophe, Krise, Krieg (3K)

**DOI:** 10.1007/s00104-026-02549-3

**Published:** 2026-08-31

**Authors:** Arnulf Gregor Willms, Sebastian Schaaf, Daniel Vallböhmer, Wolfgang E. Thasler, Aliona Wöhler, Nina Kühler, Jürgen Tepel, Lena Heidelmann, Christoph Güsgen, Andreas Westerfeld, Axel Franke, Christian Beltzer, CAMIN-Study-Group

**Affiliations:** 1 Klinik für Allgemein‑, Viszeral- und Thoraxchirurgie, Bundeswehrzentralkrankenhaus Koblenz, Rübenacher Str. 170, 56072 Koblenz, Deutschland; 2 Klinik für Allgemein- und Viszeralchirurgie & BG Klinikum Duisburg, Allgemein- und Viszeralchirurgie, Evangelisches Klinikum Niederrhein, Duisburg, Deutschland; 3 Klinik für Allgemein‑, Viszeral- und Thoraxchirurgie, Bundeswehrkrankenhaus Ulm, Ulm, Deutschland; 4 Allgemein‑, Viszeral‑, Thorax- und Minimalinvasive Chirurgie, Rotkreuzklinikum München, München, Deutschland; 5 Klinik für Allgemein‑, Viszeral- und Thoraxchirurgie, Klinikum Osnabrück, Osnabrück, Deutschland; 6 Klinik für Allgemein- und Viszeralchirurgie, Bundeswehrkrankenhaus Hamburg, Hamburg, Deutschland; 7 Klinik für Unfallchirurgie und Orthopädie, Bundeswehrkrankenhaus Ulm, Ulm, Deutschland

**Keywords:** Damage-Control-Chirurgie, Triage, Ressourcenpriorisierung, Schockraummanagement, Militärchirurgie, Damage control surgery, Triage, Resource allocation, Trauma resuscitation, Military surgery

## Abstract

**Hintergrund:**

Massenanfälle von Verletzten stellen Krankenhäuser vor erhebliche medizinische und organisatorische Herausforderungen. Charakteristisch sind eine hohe Zahl gleichzeitig eintreffender Patienten, limitierte personelle und materielle Ressourcen sowie zeitkritische Entscheidungen. Aus viszeralchirurgischer Sicht sind abdominale Verletzungen mit Blutung, Kontamination und Mehrorganbeteiligung, die eine schnelle chirurgische Versorgung und enge interdisziplinäre Zusammenarbeit erfordern, besonders relevant. Diese Verletzungsmuster finden sich typischerweise im Rahmen aktueller Terrorlagen, von Konflikten und Kriegen und sind dadurch Grundlage der Vorbereitungen für die Landes- und Bündnisverteidigung.

**Ziel der Arbeit:**

Es erfolgt die Darstellung der fachlichen, ethischen, organisatorischen und strukturellen Anforderungen an die viszeralchirurgische Versorgung im Rahmen eines Massenanfalls von Verletzten.

**Material und Methoden:**

Aus einer strukturierten Literaturanalyse wurde eine narrative Übersicht zu Katastrophenmedizin, chirurgischer Triage, Damage Control Surgery sowie organisatorischen Aspekten der chirurgischen Krisenversorgung erstellt.

**Ergebnisse:**

Zentrale Voraussetzungen für eine erfolgreiche Versorgung sind eine konsequente und lageangepasste chirurgische Triage, ein Damage-Control-orientiertes operatives Vorgehen, belastbare klinische Strukturen sowie transparente medizinethische Entscheidungsprozesse. Darüber hinaus sind standardisierte Führungs- und Kommunikationsstrukturen, interdisziplinäre Zusammenarbeit sowie regelmäßige realitätsnahe Übungen essenziell.

**Diskussion:**

Die (viszeral)chirurgische Versorgung im Massenanfall bzw. einer Belastungssituation über einen unbestimmten Zeitraum bei Konflikt oder Krieg erfordert neben operativer Expertise insbesondere Krisenkompetenz, organisatorische Resilienz und strukturierte Entscheidungsprozesse. Eine systematische Vorbereitung mit standardisierten Konzepten und Übungen ist entscheidend für eine belastbare chirurgische Krisenversorgung.

**Zusatzmaterial online:**

Die Online-Version dieses Beitrags (https://doi.org/10.1007/s00104-026-02549-3) enthält das Abkürzungsverzeichnis sowie 4 Tabellen.

## Abstract (en)

**Background:**

Mass casualty incidents with a focus on visceral surgery pose major medical and organizational challenges for hospitals. They are characterized by a large number of simultaneously admitted patients, limited personnel and material resources as well as the necessity to make time-critical surgical decisions. From a visceral surgical perspective, abdominal injuries involving severe bleeding, contamination and multiorgan involvement are particularly demanding as they require rapid surgical intervention and close interdisciplinary cooperation. This injury pattern is typically encountered in current terror situations, conflicts and wars and is therefore the foundation of the preparations for national and alliance defence.

**Objective:**

The article outlines the surgical, ethical, organizational and structural requirements for visceral surgical care in mass casualty incidents.

**Material and methods:**

Based on a structured analysis of the literature, a narrative review was performed, focusing on disaster medicine, surgical triage, damage control surgery and organizational aspects of surgical crisis management.

**Results:**

Key prerequisites for successful management include a consistent and dynamic surgical triage, damage control-oriented surgical strategies, resilient clinical structures and transparent medical ethical decision-making processes. Furthermore, standardized leadership and communication structures, interdisciplinary collaboration and regular realistic training are essential.

**Conclusion:**

Effective visceral surgical mangement during mass casualty incidents or demanding situations over an indefinite time period due to conflicts or war requires not only surgical expertise but also crisis management skills, organizational resilience and structured decision-making processes. A systematic preparation through standardized concepts and regular training is essential for sustainable surgical disaster preparedness.

Außergewöhnliche Schadenslagen stellen die Viszeralchirurgie vor besondere Herausforderungen. Neben operativer Expertise sind belastbare Strukturen, klare Entscheidungswege und eine systematische Vorbereitung erforderlich, um auch unter Krisenbedingungen handlungsfähig zu bleiben.

Aktuelle geopolitische Entwicklungen, hybride Bedrohungsszenarien sowie die Zunahme terroristischer und katastrophenbedingter Schadenslagen führen dazu, dass die Vorbereitung auf Großschadensereignisse bei 3K erneut in den Fokus der Ausrichtung und Entwicklung des Gesundheitssystems rückt.

Situationen mit einem plötzlichen Missverhältnis zwischen Patientenaufkommen und verfügbaren Ressourcen erfordern dabei ein strukturiertes, ressourcenadaptiertes Vorgehen sowie belastbare innerklinische Prozesse der Krisenbewältigung [3, 24, 30]. Viszeralchirurgische Verletzungen sind in diesen Ausnahmesituationen eine besondere Herausforderung.

Ziel dieses Beitrags ist die Darstellung wesentlicher fachlicher, organisatorischer, struktureller und ethischer Anforderungen bei der Aufrechterhaltung der viszeralchirurgischen Versorgung im MANV oder einer längerfristigen Belastungssituation bei 3K sowie die Ableitung praxisrelevanter Ansätze zur Verbesserung der chirurgischen Kriseneinsatzbereitschaft („disaster readiness“) und damit Resilienz.

## Methodik

Die Erstellung und Darstellung des Beitrags orientierte sich an den Empfehlungen der SANRA(Scale for the Assessment of Narrative Review Articles)-Kriterien für narrative Übersichtsarbeiten. Für die Erstellung dieses Übersichtsartikels erfolgte eine strukturierte Literaturrecherche in der Datenbank PubMed. Berücksichtigt wurden aktuelle Leitlinien, Übersichtsarbeiten, Positionspapiere sowie ausgewählte Originalarbeiten zu den Themen Massenanfall von Verletzten (MANV), Katastrophenmedizin, Triage, Damage Control Surgery, penetrierende abdominelle Verletzungen, Notfalllaparotomie und Open-Abdomen-Therapie. Die Recherche erfolgte unter Verwendung deutscher und englischer Suchbegriffe, darunter „MANV“, „MASCAL“, „mass casualty incident“, „disaster surgery“, „damage control surgery“, „triage“ sowie Kombinationen mit „visceral surgery“, „hospital management“ und „organization“. Berücksichtigt wurden überwiegend Publikationen der vergangenen 10 Jahre.

## Ergebnisse

Die Organisation der chirurgischen Versorgung im MANV lässt sich in abgestufte Eskalationsphasen einteilen, die sich an Ressourcenverfügbarkeit und infrastruktureller Belastung orientieren [14]. Die Maßnahmen der chirurgischen Versorgung im MANV lassen sich entsprechend an eine kompensierte, gefährdete und dekompensierte Krisenversorgung anpassen (Supplement Tab. 3). Mit zunehmender Ressourcenknappheit erfolgt eine stufenweise Fokussierung von einer möglichst standardnahen Versorgung hin zu ausschließlich lebensrettenden Maßnahmen der Blutungs- und Kontaminationskontrolle.

### Fachliche Anforderungen der Viszeralchirurgie in MANV und Krise

#### Versorgungsprinzipien der chirurgischen Krisenversorgung

Die operative Versorgung im MANV und bei 3K folgt den Prinzipien einer ressourcenadaptierten Chirurgie. Während in frühen Phasen dringliche onkologische oder rekonstruktive Eingriffe teilweise noch durchgeführt werden können, beschränkt sich die chirurgische Therapie im weiteren Verlauf zunehmend auf unmittelbar lebensrettende Maßnahmen [3].

Zentrale operative Ziele sind [8, 14]:

- rasche Blutungskontrolle,
- Minimierung intraabdomineller Kontamination,
- Verkürzung der Operationsdauer sowie
- eine möglichst geringe Ressourcenbindung.

Definitive Rekonstruktionen werden zugunsten einer abgestuften, zeitverzögerten Rekonstruktion, der sog. „staged reconstruction“ bewusst zurückgestellt. Second-Look-Operationen und die verzögerte Anlage von Anastomosen sind integraler Bestandteil etablierter Damage-Control-Konzepte und können bei persistierender Instabilität oder dekompensierter Versorgungslage geplant hinausgeschoben werden. Entscheidend ist ein dynamischer Abgleich zwischen physiologischem Zustand des Patienten und aktueller Ressourcenverfügbarkeit im Rahmen der kontinuierlichen tagesaktuellen Lagebeurteilung und Re-Evaluation.

#### Damage Control Surgery und Tactical Abbreviated Surgical Care

Die Damage Control Surgery (DCS) stellt das zentrale operative Konzept der modernen Traumachirurgie dar [3, 18]. Das von Stone [48] 1983 und Rotondo [44] 1993 geprägte Konzept beschreibt eine abgekürzte, zweizeitige Operation bei vital bedrohten Patienten im hämorrhagischen Schock, jedoch im individualmedizinischen Kontext zur Reduktion der Systembelastung des Organismus [40, 53].

Die Damage Control Surgery stellt das zentrale operative Konzept der modernen Traumachirurgie dar

Indikationen zur DCS können der physiologische Status (z. B. Koagulopathie, Azidose, Hypothermie, Hypokalzämie) und/oder die Verletzungsschwere (z. B. schwere Blutung, schweres Lebertrauma in Kombination mit weiteren viszeralen Verletzungen) sein [34, 41].

**Abb. 1:**
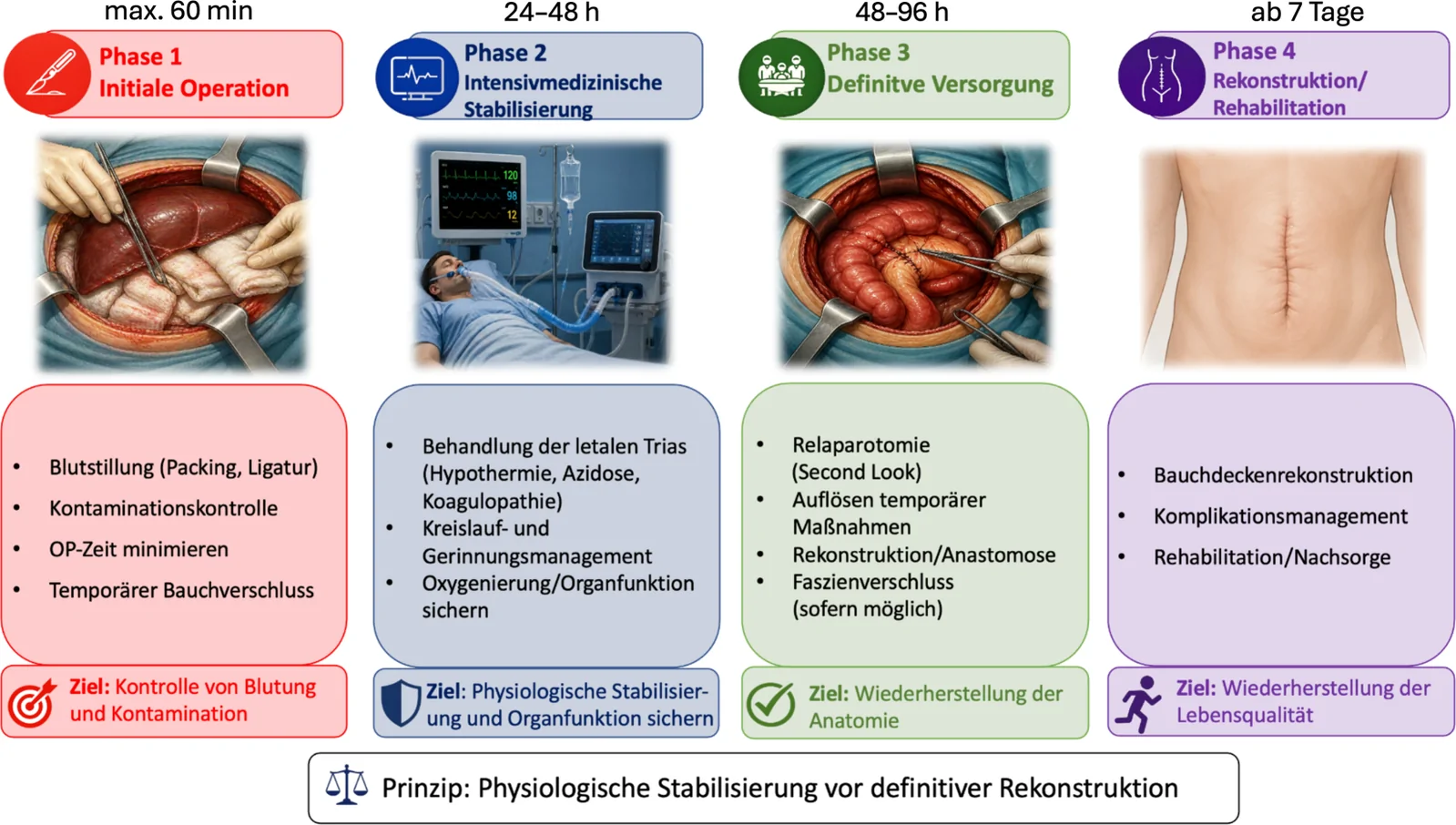
Schematische Darstellung der 4 Phasen der Damage-Control-Chirurgie im Massenanfall von Verletzten: initiale operative Blutungs- und Kontaminationskontrolle, intensivmedizinische Stabilisierung, definitive chirurgische Versorgung sowie spätere Rekonstruktion und Rehabilitation. (Erstellt mit ChatGPT 5)

Klassische Bestandteile sind Packing, temporäre Gefäßkontrolle, Resektion ohne primäre Anastomose sowie temporäre Bauchdeckenverschlüsse [8, 19]. Die Abb. 1 stellt die Sequenz der DCS und die therapeutische Zielsetzung dar.

Hiervon abzugrenzen ist das Konzept der Tactical Abbreviated Surgical Care (TASC). Während DCS primär mit der drohenden oder manifesten physiologischen Erschöpfung oder Verletzungsschwere des einzelnen Patienten begründet wird, beschreibt TASC eine ressourcenadaptierte chirurgische Strategie zur Maximierung der Gesamtversorgungskapazität und des Überlebens möglichst Vieler unter Krisenbedingungen (Supplement Tab. 1) [14].

TASC verfolgt damit einen systemorientierten Ansatz und berücksichtigt neben dem individuellen Patientenstatus insbesondere die aktuelle Operationskapazität, Materialverfügbarkeit, Intensivressourcen sowie die Gesamtdynamik des MANV. Definitive Rekonstruktionen werden hierbei bewusst zugunsten kurzer Eingriffe mit geringer Ressourcenbindung zurückgestellt.

#### „Open Abdomen“ und „staged reconstruction“

Der temporäre Bauchdeckenverschluss ermöglicht eine schnelle Beendigung der Primäroperation, reduziert Operationszeiten, antizipiert postoperative Herausforderungen und erlaubt eine geplante Re-Evaluation nach physiologischer Stabilisierung [42]. Gleichzeitig schafft das Open-Abdomen-Konzept Flexibilität für wiederholte Re-Laparotomien im Rahmen von Second-Look-Strategien. Geplante Second-Look-Laparotomien erfolgen im klassischen Damage-Control-Konzept typischerweise innerhalb von 24–48 h. Unter Bedingungen persistierender Lageinstabilität oder ausgeprägter Ressourcenknappheit erscheint im MANV-Kontext jedoch eine Verzögerung auf bis zu 72–96 h vertretbar.

Nach Stabilisierung auf der Intensivstation erfolgt nach 24–48 h die programmierte Second-Look-Operation mit De-Packing und intestinaler Rekonstruktion [16]. Die Faszie sollte im Rahmen der Second-Look-Operation, wenn möglich, sekundär verschlossen werden. Bei erforderlicher Weiterführung der offenen Abdominalbehandlung sollten zur Reduktion von Morbidität und Mortalität strukturierte und evidenzbasierte Algorithmen empfohlen werden, die die zentralen Therapieelemente Faszientraktion, Viszeralschutz und kontrollierte VAC-Therapie integrieren [6, 54, 55, 57, 58].

Intestinale Anastomosen zeigen unter offener Abdominaltherapie keine erhöhte Insuffizienzrate, sodass der Entschluss zur Rekonstruktion unabhängig von einem möglichen Abdomenverschluss getroffen werden sollte [7].

Entscheidend für die spätere Rekonstruktion der Bauchdecke und die Entstehung der ventralen Hernie erscheint die Strategie zur Bauchdecken- und Faszienbehandlung [58]. Die offene Abdominalbehandlung wird grundsätzlich so lange aufrechterhalten, bis ein sekundärer Faszienverschluss möglich ist. Bei nicht mehr erzielbarem sekundärem Faszienverschluss wird die Vakuumtherapie am Abdomen mittels Skin-only-Verschluss beendet – mit rekonstruktiver Versorgung der programmierten ventralen Hernie im Verlauf [28, 45].

#### Operative Schlüsselkompetenzen

Die viszeralchirurgische Versorgung im MANV erfordert spezifische operative Kernkompetenzen, die im zivilen Klinikalltag zunehmend seltener trainiert werden (Supplement Tab. 2). Daher gewinnt das Konzept der „readiness through routine“ an Bedeutung: Trauma- und MANV-relevante operative Teilschritte sollten gezielt in den klinischen Alltag und die chirurgische Ausbildung integriert werden. Insbesondere komplexe viszeralonkologische Eingriffe können zum Erhalt dieser Kompetenzen beitragen.

### Strukturelle Anforderungen

#### Infrastruktur und skalierbare Behandlungskapazitäten – „surge capacity“

Eine resiliente viszeralchirurgische Versorgung im Rahmen eines MANV bei 3K erfordert belastbare, redundant abgesicherte und flexibel skalierbare Krankenhausstrukturen [25, 30, 50, 51]. Neben digital unterstützten Prozessen müssen im Sinne einer resilienten Krisenvorbereitung auch Low-Tech-Redundanzen vorgehalten werden.

Kurzfristige Mobilisierung operativer und intensivmedizinischer Kapazitäten

Hierzu zählen papierbasierte Dokumentationssysteme, analoge Kommunikationswege sowie robuste Ausfallkonzepte für Strom‑, Wasser- und IT-Infrastruktur [21]. Zentrale Voraussetzung ist die Fähigkeit zur kurzfristigen Mobilisation operativer und intensivmedizinischer Kapazitäten im Sinne einer bedarfsgerecht optimierten „surge capacity“ [14, 43]. Hierzu zählen aktivierbare Operationskapazitäten, ausreichende intensivmedizinische Ressourcen sowie belastbare Führungs- und Kommunikationsstrukturen im Rahmen der Krankenhausalarm- und Einsatzplanung (KAEP) [3, 8, 14, 51].

Wichtig ist die Fähigkeit, vorhandene Räume bedarfsgerecht als Operationsbereich umzufunktionieren. Die dafür notwendige Grundausstattung umfasst: eine geeignete Lagerungsmöglichkeit, einen funktionsfähigen Narkosearbeitsplatz, ausreichende Beleuchtung – notfalls durch mobile OP-Leuchten oder Kopflampen –, viszeralchirurgische Basisinstrumente, sterile Mehrfachabdeckungen, ausreichendes Nahtmaterial sowie Optionen zum temporären Bauchdeckenverschluss [3, 14, 19, 35, 42].

Unter prolongierten Krisenbedingungen kann auch die Nutzung alternativer OP-Bereiche oder temporärer chirurgischer Versorgungseinheiten erforderlich werden [51]. Raumlufttechnische Anforderungen müssen lage- und ressourcenabhängig bewertet werden. In Extremsituationen kann eine Abweichung von regulären OP-Standards akzeptabel sein, sofern lebensrettende operative Maßnahmen andernfalls nicht zeitgerecht durchgeführt werden können [3, 14]. Im Extremfall kann dies bedeuten, dass z. B. auf wiederverwendbare OP-Abdeckungen zurückgegriffen werden muss und Anastomosen wieder von Hand genäht werden.

#### Materialvorhaltung und operative Ausstattung

Neben spezialisierten Damage-Control-Sets sollten einfache, robuste und reproduzierbar einsetzbare Instrumentensysteme verfügbar gemacht werden. Hierzu zählen standardisierte Notfallsiebe, beispielsweise die „Berliner Notfallsiebe“, sowie ausreichende Vorräte an Nahtmaterial als Alternative zu staplerbasierten Verfahren [3].

Gleichzeitig müssen Blutprodukte, Verbrauchsmaterialien und intensivmedizinische Ressourcen für definierte Zeiträume verfügbar gehalten werden. Aktuelle Untersuchungen zeigen jedoch relevante Defizite in Materialvorhaltung, personeller Freistellung und struktureller Vorbereitung deutscher Krankenhäuser auf [14, 32, 50].

Die Vorhaltung von Material muss darüber hinaus eine ausreichende Durchhaltefähigkeit unter prolongierten Krisenbedingungen gewährleisten. Aktuelle Konzepte der Krankenhausresilienz und KRITIS-Planung orientieren sich hierbei häufig an einer Mindestautarkie von 72 h, wobei Umfang und Dauer der Vorhaltung von Versorgungsstufe, regionalem Versorgungsauftrag und erwartetem Patientenaufkommen abhängig sind [26, 31].

### Organisatorische Anforderungen

#### Krankenhausalarm- und Einsatzplan (KAEP)

Die viszeralchirurgische Versorgung im MANV erfordert eine organisatorische Einbindung in die klinische Führungsstruktur. Grundlage ist die Integration viszeralchirurgischer Anforderungen in den Krankenhausalarm- und Einsatzplan (KAEP) [8, 49]. Hierzu gehören insbesondere die Definition viszeralchirurgischer Verantwortlichkeiten, operative Priorisierungsstrategien, standardisierte Damage-Control- und TASC-Abläufe sowie die strukturierte Einbindung relevanter Schnittstellen wie Gefäßchirurgie, Urologie, Gynäkologie, Anästhesie, Intensivmedizin, Radiologie und Blutbank [27, 51].

Neben der Aktivierung des KAEP ist die frühzeitige Einbindung viszeralchirurgischer Entscheidungsträger wichtig, da intraabdominelle Verletzungen mit hoher Dringlichkeit, erheblicher Ressourcenbindung und komplexen Priorisierungsentscheidungen verbunden sind [8]. Hier muss institutionell im KAEP sichergestellt werden, dass die Bedürfnisse aller operativ zu versorgenden Patienten (viszeralchirurgisch, wie z. B. traumatologisch) durch die für diese Aufgabe etablierte Führungsfunktion (z. B. den „zentralen operativen Notfallkoordinator“ [ZONK]) berücksichtigt, priorisiert und in enger Abstimmung mit OP-Koordination, Intensivmedizin und klinischem Krisenstab verbindlich umgesetzt werden.

#### Operative Priorisierung und Ressourcennutzung

In MANV-Situationen erfolgt die operative Entscheidungsfindung konsequent ressourcenadaptiert und weicht bewusst von individualmedizinischen Standards ab (Supplement Tab. 3).

#### Interdisziplinäre Zusammenarbeit und Ressourcennutzung

Eine resiliente chirurgische Krisenversorgung erfordert eine interdisziplinäre Erweiterung operativer Kapazitäten. Fachdisziplinen mit überlappenden Kompetenzen in der Notfalllaparotomie, insbesondere Gefäßchirurgie, Gynäkologie und Urologie, können definierte Anteile standardisierter Damage-Control- oder explorativer Eingriffe übernehmen. Ziel ist eine ressourcen- und kompetenzbasierte Organisation, bei der viszeralchirurgische Kernkompetenzen gezielt für komplexe Rekonstruktionen reserviert werden [13, 14].

#### Patientenallokation und Versorgungskoordination

Ein weiterer zentraler organisatorischer Aspekt ist die Steuerung der Patientenflüsse. Grundlage ist die Aktivierung des KAEP. Die Koordination zwischen Eingangssichtung, Behandlungsbereichen (Schockraum), OP, Intensivstation und Normalstation muss zentral gesteuert werden, um Verzögerungen in der operativen Versorgung zu vermeiden [27]. Ergänzend sind regionale Verlegungsstrategien, softwaregestützte Echtzeitübersichten relevanter Ressourcen sowie redundante Kommunikations- und Dokumentationsstrukturen erforderlich, um die Versorgung auch bei IT-Ausfällen oder prolongierten Krisenlagen aufrechtzuerhalten und die Business Continuity kritischer Krankenhausfunktionen sicherzustellen [21].

### Medizinethische Anforderungen und Ressourcenallokation

Aktuelle Empfehlungen zur medizinischen Entscheidungsfindung in Gesundheitskrisen betonen, dass in Krisensituationen individuelle Therapieziele gegenüber einer gerechten und transparenten Ressourcenallokation zurücktreten können [4, 5, 9, 10, 22, 23]. Grundlage sind standardisierte, nachvollziehbare und diskriminierungsfreie Entscheidungsprozesse. Zentrale Prinzipien sind die Gleichbehandlung aller Patienten, die Priorisierung nach Erfolgsaussicht und Ressourcenverbrauch, die kontinuierliche Re-Evaluation sowie die ethische Gleichstellung von Therapiebegrenzung und Therapieabbruch unter Krisenbedingungen.

Hieraus ergeben sich erhebliche psychische Belastungen für Behandlungsteams („moral distress“), insbesondere im Rahmen von Therapieabbrüchen oder Priorisierungsentscheidungen [10, 37]. Daher sind ethische Entscheidungsstrukturen und vorbereitete klinische Handlungskonzepte integraler Bestandteil der Krisenvorbereitung [8, 10, 22, 27]. Maßnahmen ohne relevante Erfolgsaussicht oder mit unverhältnismäßiger Ressourcenbindung besitzen im ausgeprägten MANV keinen Stellenwert [11, 13, 14, 30].

#### Chirurgische Triage-Systeme

Eingangssichtung, Indikationsstellung, Priorisierung, Re-Evaluation und ethische Entscheidungsfindung sind zentrale Elemente der Versorgung im MANV. Im Vordergrund steht die Priorisierung mit dem Ziel, unter Wahrung von Transparenz, Gleichbehandlung und Menschenwürde den größtmöglichen Nutzen für möglichst viele Patienten zu erzielen [22].

Zur strukturierten chirurgischen Priorisierung wurden verschiedene Algorithmen entwickelt. Die von der World Society of Emergency Surgery entwickelte TACS(Timing of Acute Care Surgery)-Klassifikation ermöglicht die operative Priorisierung anhand definierter Zeitkategorien („immediate“, „urgent“, „delayed“, „elective“) [29]. Immediate-Patienten werden bevorzugt einer Damage-Control-Strategie zugeführt, während weniger zeitkritische Eingriffe zurückgestellt werden können.

Ergänzend dient die META-Methode (Prehospital Advanced Triage Method) der frühzeitigen präklinischen Identifikation chirurgisch relevanter Hochrisikopatienten anhand definierter Verletzungsmuster und hämodynamischer Kriterien [1, 49].

#### Dynamische Entscheidungsprozesse

**Abb. 2:**
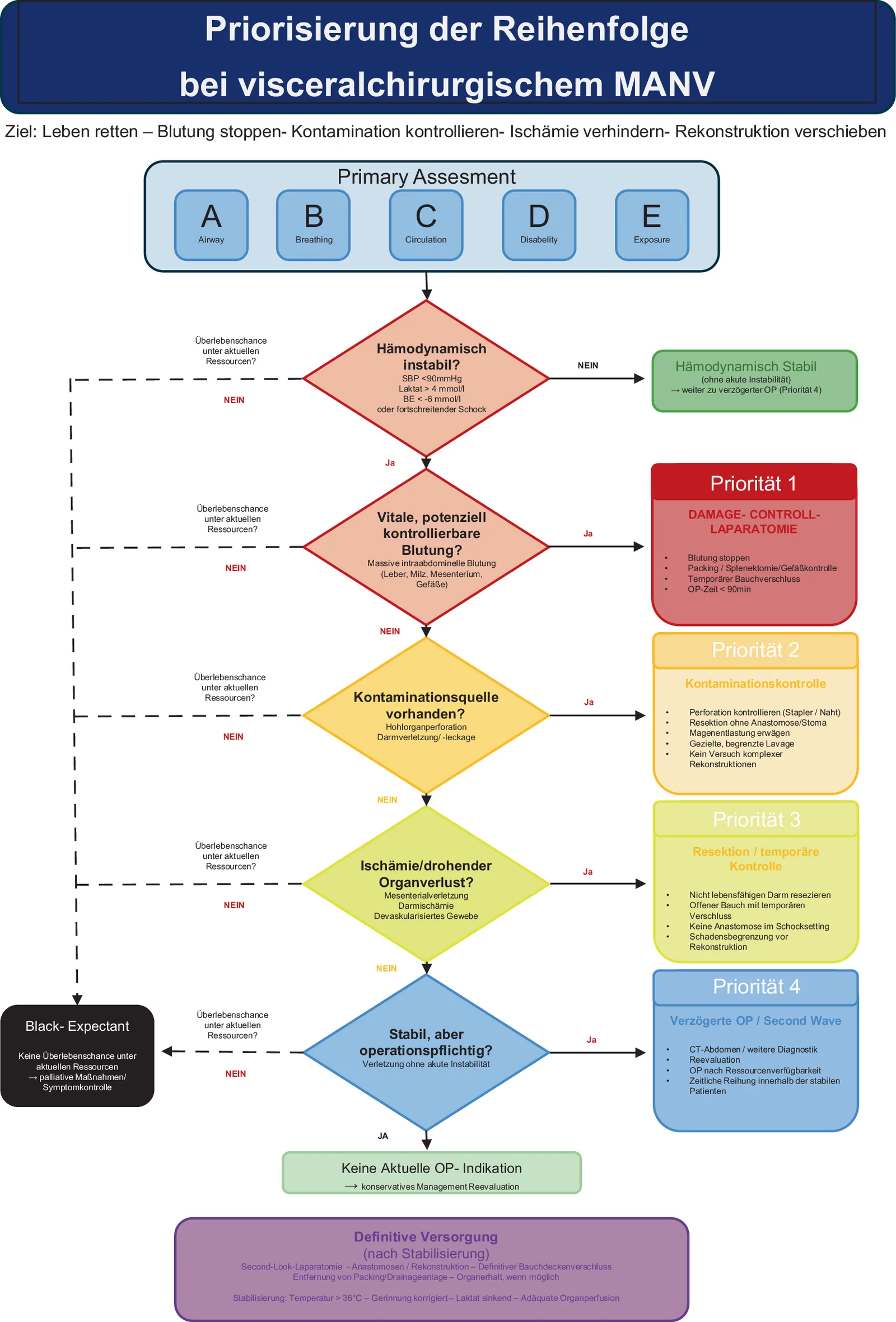
Schematische Darstellung eines synthetisierten viszeralchirurgischen Triage- und Priorisierungskonzepts im Rahmen eines Massenanfalls von Verletzten (MANV). Die Abbildung kombiniert etablierte Prinzipien der präklinischen Hochrisikoselektion mittels META-Methode (Prehospital Advanced Triage Method) [1] mit der innerklinischen operativen Priorisierung nach der TACS-Klassifikation (Timing of Acute Care Surgery, WSES) [29]. (© die Autoren)

Die Verlaufsbeurteilung/Re-Triage (nach der Eingangssichtung und der Initialbehandlung in den dafür vorgesehenen Behandlungsbereichen) im MANV ist kein statischer Vorgang, sondern ein kontinuierlicher Entscheidungsprozess unter sich im Verlauf verändernden personellen, materiellen und infrastrukturellen Bedingungen. Die operative Priorisierung muss daher fortlaufend an Patientenaufkommen, Ressourcenverfügbarkeit und Gesamtlage angepasst werden. Entscheidend bleibt hierbei die Balance zwischen medizinischer Erfolgsaussicht, ethischer Vertretbarkeit und maximaler Versorgungskapazität [15, 20, 22]. Diese Faktoren bestimmen die ärztliche Indikationsstellung für weitere operative Maßnahmen. Ein schematischer Überblick über die viszeralchirurgische Verlaufsbeurteilung/Re-Triage und operative Priorisierung im MANV unter Integration von META-, TACS- und Damage-Control-Prinzipien ist in Abb. 2 dargestellt.

### Vorbereitung – Ausbildung und Training

Die Umsetzung strukturierter Versorgungsstrategien für den MANV bei 3K erfordert ein hohes Maß an standardisiertem Training und regelmäßiger interdisziplinärer Übung. Ziel ist die Entwicklung individueller operativer Kompetenz sowie team- und systembezogener Handlungssicherheit unter Zeitdruck und Ressourcenknappheit.

#### Standardisierte Kursformate

Etablierte Ausbildungskonzepte bilden die Grundlage der chirurgischen Krisenvorbereitung. Das American College of Surgeons-Programm ATLS® (Advanced Trauma Life Support) vermittelt die Primärversorgung Schwerverletzter nach dem xABCDE-Schema und verbessert nachweislich Wissen, Entscheidungsfindung und organisatorische Kompetenzen im Traumamanagement [13]. Die Prinzipien sind auch unter MANV-Bedingungen anwendbar. Ergänzend fokussieren operative Traumakurse wie DSTC® (Definitive Surgical Trauma Care), TDSC® (Tactical and Disaster Surgical Care) oder ACT!® (Advanced Surgical Skills for Exposure in Trauma) auf Damage-Control-Chirurgie, Notfalllaparotomien sowie operative Entscheidungsstrategien unter Ressourcenlimitation und Zeitdruck [2, 13, 18, 27, 47]. Insbesondere die Versorgung penetrierender und explosionsbedingter Verletzungen stellt im zivilen Setting eine seltene, aber hochrelevante Kompetenz dar [11, 12, 14].

#### Simulation und interdisziplinäres Teamtraining

MANV-spezifische Trainingsprogramme vermitteln neben medizinisch-technischen Fertigkeiten insbesondere Triage-Algorithmen, Ressourcensteuerung sowie Führungs- und Kommunikationsstrukturen [17]. High-Fidelity-Simulationen, interdisziplinäre Schockraumtrainings und MANV-Übungen verbessern nachweislich Entscheidungsfindung, Teamkommunikation und klinische Handlungssicherheit unter Krisenbedingungen [38].

Zentrale Trainingsinhalte sind:

- strukturierte Schockraumversorgung und Damage-Control-Prinzipien,
- Management intraabdomineller Blutungen und Kontaminationen,
- operative Priorisierung unter Ressourcenknappheit,
- Re-Triage und dynamische Entscheidungsprozesse,
- Crew-Ressource-Management (CRM) und Führungsstrukturen,
- Integration ethischer Entscheidungsprinzipien in klinische Abläufe.

#### Institutionelle Krisenvorbereitung

Eine nachhaltige viszeralchirurgische Einsatzbereitschaft basiert auf standardisierten Prozessen, klinischer Erfahrung und regelmäßigen Übungen. Neben interdisziplinärer Zusammenarbeit und ressourcenadaptierten Entscheidungsprozessen müssen insbesondere traumatologische und notfallchirurgische Kernkompetenzen wie Notfalllaparotomie, Blutungskontrolle, Open-Abdomen-Verfahren und Damage-Control-Strategien gezielt im klinischen Alltag erhalten werden [52, 56]. Hierzu ist es sinnvoll, Trauma- und MANV-relevante operative Teilschritte auch im Rahmen elektiver Eingriffe mitzudenken und gezielt für Ausbildungszwecke zu nutzen. Dieses Konzept der „readiness through routine“ ermöglicht den Erhalt chirurgischer Handlungssicherheit trotz geringer klinischer Exposition gegenüber penetrierenden oder explosionsbedingten Verletzungen [33, 36, 39, 46].

Mit dem Curriculum „Notfall- und Katastrophenchirurgie“ hat die Akademie der Unfallchirurgie (AUC) ein strukturiertes Weiterbildungskonzept für MANV-, Terror- und Katastrophenszenarien etabliert. Ergänzend entwickelt die CAMIN der Deutschen Gesellschaft für Allgemein- und Viszeralchirurgie (DGAV) eine fachspezifische Qualifikation für die viszeralchirurgische Krisenversorgung. Eine Übersicht zentraler Kriterien zur Bewertung der Krisenresilienz und MANV-Vorbereitung ist im Supplement in Tab. 4 dargestellt.

## Fazit für die Praxis

- Die viszeralchirurgische Versorgung im MANV sowie im Kontext von Krise, Katastrophe und Krieg erfordert standardisierte Damage-Control- und TASC-Strategien, klare Führungsstrukturen und konsequente Ressourcenpriorisierung.
- Krankenhausalarm- und Einsatzpläne (KAEP) sollten fachspezifische viszeralchirurgische Abläufe, Re-Triage-Prozesse und operative Eskalationsstufen verbindlich definieren.
- Eine resiliente Krisenversorgung setzt belastbare Materialvorhaltung, skalierbare OP- und Intensivkapazitäten sowie interdisziplinäre Zusammenarbeit mit Gefäßchirurgie, Urologie, Gynäkologie und weiteren operativen Disziplinen voraus.
- Regelmäßige simulationsbasierte Trainings, standardisierte Kursformate und strukturierte Fähigkeitsanalysen sind essenziell für die chirurgische Einsatzbereitschaft und die sichere Umsetzung von DCS- und TASC-Konzepten (notwendiger Kompetenzerwerb in notfallchirurgischen Fähigkeiten).
- Die Vorbereitung auf MANV- und MASCAL-Szenarien erfordert eine kontinuierliche Analyse von Resilienz, Vulnerabilität, Fähigkeit der Krisenbewältigung und Aufwuchsfähigkeit der eigenen Klinikstrukturen.

## Supplementary Information

ESM1: Zusatzmaterial 1

## Data Availability

Alle dieser Arbeit zugrunde liegenden Daten sind in diesem Artikel enthalten.
