# Supplementary material for: Visceral surgery in mass casualty incidents due to disasters, crises and war
Source: Chirurgie (Heidelb). 2026 Aug 31;97(10):777–85. [Article in German] doi: 10.1007/s00104-026-02549-3 (PMC13601115; doi:10.1007/s00104-026-02549-3)
Supplement: Supplementary file 1 [file 104_2026_2549_MOESM1_ESM.pdf]

Supplement: Viszeralchirurgie im Massenanfall bei Katastrophe, Krise, Krieg (3K)

Abkürzungsverzeichnis

ACT!®	Advanced Surgical Skills for Exposure in Trauma
ATLS®	Advanced Trauma Life Support
DCS	Damage Control Surgery
DSTC®	Definitive Surgical Trauma Care
eFAST	extended Focused Assessment with Sonography for Trauma
KAEP	Krankenhausalarm- und Einsatzplanung
MANV	Massenanfall an Verletzen
MASCAL	mass casualty incident
META	Prehospital Advanced Triage Method
SANRA-Kriterien	Scale for the Assessment of Narrative Review Articles
SOP	Standard Operating Procedure
TASC	Tactical Abbreviated Surgical Care
TDSC®	Tactical and Disaster Surgical Care

Supplement-Tabelle 1 Gegenüberstellung der Konzepte Damage-Control-Surgery (DCS) und Tactical Abbreviated Surgical Care (TASC). Während DCS primär patientenbezogen durch physiologische Instabilität oder Verletzungsschwere begründet ist, beschreibt TASC eine ressourcenadaptierte operative Strategie unter Bedingungen personeller oder materieller Ressourcenknappheit im Rahmen eines Massenanfalls oder militärischer Einsatzlagen.

**Aspekt**	**Damage Control Surgery (DCS)**	**Tactical Abbreviated Surgical Care (TASC)**
Grundprinzip	Patientenbezogene fokussierte, abgekürzte Chirurgie	Ressourcenadaptierte taktische Chirurgie
Hauptindikation	Physiologische Instabilität und/oder Verletzungsschwere	Personelle oder materielle Ressourcenknappheit
Fokus	Überleben des einzelnen Patienten	Maximierung der Gesamtversorgungskapazität
Operative Strategie	Blutungs- und Kontaminationskontrolle mit geplanter Rekonstruktion	Konsequente Verkürzung von Operationsdauer und Ressourcenverbrauch mit Ziel Überleben ermöglichen
Rekonstruktionen	Möglichst nach Stabilisierung	Bewusst verzögert oder vereinfacht
Typische Maßnahmen	Packing, temporärer Bauchverschluss, staged reconstruction	Vereinfachte Verfahren, Verzicht auf zeitaufwendige Rekonstruktionen
Klinischer Kontext	Schwerverletztenversorgung	MANV-/MASCAL-/Kriegsszenarien
Konsequenz	„Physiology before anatomy“	„Do the most for the most“
Mögliche Konsequenzen	Temporäre funktionelle Einschränkungen	Akzeptanz funktioneller Nachteile zugunsten der Gesamtlage (z. B. Splenektomie statt milzerhaltender Versorgung)

Supplement-Tabelle 2 Übersicht zentraler technischer Fertigkeiten und operativer Manöver für Damage-Control-Surgery (DCS) und Tactical Abbreviated Surgical Care (TASC). Die aufgeführten Maßnahmen dienen primär der schnellen Blutungs- und Kontaminationskontrolle im Rahmen zeitkritischer viszeralchirurgischer Notfalleingriffe unter individualmedizinischen beziehungsweise ressourcenlimitierten Krisenbedingungen.

**Bereich**	**Technische Fertigkeiten und Manöver**
**Traumazugang und Exploration**	Mediane Traumalaparotomie (ggf. mit Erweiterungen), strukturierte Exploration des Abdomens
**Blutungskontrolle Leber**	Lebermobilisation (v. a. rechts), Leberpacking, Pringle-Manöver
**Retroperitoneale Exposition**	Cattell-Braasch-Manöver (rechtsseitige viszerale Rotation), Mattox-Manöver (linksseitige viszerale Rotation)
**Gefäßkontrolle und Gefäßchirurgie**	Gefäßnaht (z. B. Vena cava inferior), inframesokolisches Klemmen der Aorta abdominalis, temporäre Gefäßkontrolle
**Milz- und Pankreaschirurgie**	Splenektomie, ggf. mit Pankreasschwanzresektion, Zugang zur Bursa omentalis
**Darm- und Kontaminationskontrolle**	Enterale Diskontinuitätsresektionen, Stapling-Verfahren, Verzicht auf primäre Anastomosen
**Retroperitoneale Organverletzungen**	Zugang zur Niere beidseits, Nephrektomie
**Open-Abdomen-Verfahren**	Temporärer Bauchdeckenverschluss (Open Abdomen/VAC)
**Thorakoabdominelle Notfallverfahren**	Anterolaterale Notfallthorakotomie links mit aortalem Cross-Clamping und Perikarderöffnung

**Übergeordnete operative Kompetenzen**	Schnelle Blutungskontrolle, Damage-Control-Prinzipien, Operationsverkürzung unter Zeitdruck, ressourcenadaptiertes Operieren im Sinne von DCS/TASC

Supplement-Tabelle 3: Merkmale verschiedener Stufen der Krisenversorgung; MIC = minimalinvasive Chirurgie; TASC = tactical abbreviated surgical care

	Beschreibung	Elektive Chirurgie	Onkologische Eingriffe	Materialressourcen	Behandlungsmodus
**Kompensierte Krisenversorgung**	Neben der Notfallversorgung können dringliche oder onkologische Eingriffe teilweise weiterhin durchgeführt werden, während elektive Operationen ausgesetzt werden. Hierbei ist es das Ziel durch Umverteilung, Umorganisation, Umwidmung noch für möglichst alle Verletzten und Notfallpatienten ein der individualmedizinischen Standardversorgung vergleichbares Ergebnis über den gesamten Zeitraum der Behandlung abzusichern.	nein	ja	nicht eingeschränkt	MIC und offen
**Gefährdete Krisenversorgung**	Die chirurgische Versorgung konzentriert sich überwiegend auf zeitkritische Notfalleingriffe und Damage-Control-Verfahren bei zunehmender Ressourcenknappheit. Das abschließende Behandlungsergebnis kann vom	nein	nur nicht aufschiebbare Tumorchirurgie	beginnende Einschränkung	MIC und offen

	individualmedizinisch erreichbaren Standard abweichen.				
**Dekompensierte Krisenversorgung**	Operative Maßnahmen werden auf unmittelbar lebensrettende Eingriffe zur Blutungs- und Kontaminationskontrolle reduziert. Definitive Rekonstruktionen treten zugunsten einer maximalen Notfallversorgungskapazität in den Hintergrund.	nein	nein	erhebliche Einschränkung	offen, TASC

Supplement-Tabelle 4 Checkliste viszeralchirurgische Resilienz im MANV. Checkliste zentraler struktureller, organisatorischer und personeller Anforderungen zur Bewertung der viszeralchirurgischen Resilienz und Krisenvorbereitung im Rahmen eines Massenanfalls von Verletzten (MANV). Die Tabelle umfasst Aspekte der Alarm- und Einsatzplanung, operativen Kapazität, Materialvorhaltung, interdisziplinären Zusammenarbeit, Triage sowie Ausbildung und Qualitätssicherung.

**Bereich**	**Prüffrage**
Alarm- und Einsatzplanung	Existiert ein schriftlich etablierter und regelmäßig aktualisierter KAEP mit viszeralchirurgischen SOPs?
Führungsstruktur	Sind viszeralchirurgische Führungsrollen und Entscheidungswege eindeutig definiert?
Operative Kapazitäten	Können zusätzliche OP-Kapazitäten kurzfristig aktiviert oder umgewidmet werden?
Damage-Control-Fähigkeit	Stehen Damage-Control- und Open-Abdomen-Setups jederzeit verfügbar bereit?
Materialvorhaltung	Besteht eine definierte Vorhaltung essenzieller Materialien (Nahtmaterial, Mehrfachabdeckungen, Notfallsiebe, Blutprodukte)?
Durchhaltefähigkeit	Ist eine autarke chirurgische Versorgung über mindestens 72 h oder ggfs. länger gesichert?

Interdisziplinäre Zusammenarbeit	Sind Schnittstellen zu Gefäßchirurgie, Urologie, Gynäkologie, Intensivmedizin und Radiologie definiert?
Patientenflusssteuerung	Bestehen standardisierte Prozesse für OP-, Intensiv- und Verlegungskoordination?
Triage	Sind Zuständigkeiten für Eingangssichtung, chirurgische Triage, und Reevaluation klar festgelegt?
Ausbildung	Werden regelmäßige Schockraum-, MANV- und simulationsbasierte Trainings durchgeführt?
Kompetenzmanagement	Existieren Fähigkeitsmatrizen zur strukturierten Selbst- und Fremderfassung operativer Kompetenzen?
Qualitätssicherung	Werden strukturierte Debriefings, SOP-Updates und Qualitätsindikatoren genutzt?
